# A novel computational tool for tracking cancer energy hijacking from immune cells

**DOI:** 10.1002/ctm2.1533

**Published:** 2024-01-09

**Authors:** Hongyi Zhang, Bo Li

**Affiliations:** ^1^ Center for Computational and Genomic Medicine The Children's Hospital of Philadelphia Philadelphia Pennsylvania USA; ^2^ Department of Pathology and Laboratory Medicine University of Pennsylvania Philadelphia Pennsylvania USA

**Keywords:** Deconvolution, Mitochondrial Transfer, single‐cell genomics, Tumor‐immune Interaction

## Abstract

Recent studies revealed a new biological process that malignant cancer cells hijack mitochondria from nearby T cells, providing another potential mechanism for immune evasion. We further confirmed this process at the single‐cell genomic level through MERCI, a novel algorithm for tracking mitochondrial (MT) transfer. Applied to human cancer samples, MERCI identified a new cancer phenotype linked to MT hijacking, correlating with rapid tumour proliferation and poor patient survival. This discovery offers insights into the limitations of current cancer immunotherapies and suggests new therapeutic avenues targeting MT transfer to enhance cancer treatment efficacy.

## 1 A NOVEL TUMOUR‐IMMUNE INTERACTION: MT TRANSFER BETWEEN CANCER AND T CELLS

As pivotal effectors of the immune system, T cells are vigilantly patrolling our bodies to target and eliminate diseased cells. However, T cells infiltrating into the tumour microenvironment usually encounter metabolic stresses that ultimately cause T cell dysfunction.^1^ This process is generally considered associated with the upregulation of immune inhibitory receptors, such as PD‐1, LAG3, TIM‐3 and CTLA‐4. Previous studies have shown that cancer cells can capture mitochondria, the energy powerhouses of eukaryotic cells, from the surrounding microenvironment to support their metabolic demands.^2^, ^3^ More recently, Saha et al.^4^ found that, under cell culture conditions, some cancer cells can extend long nanotube (NT) protrusions into the T cells and sucks mitochondria from these nearby T cells, a phenomenon observed for the first time. This process not only aids tumour survival but also impairs T‐cell function. A better understanding of the molecular mechanisms of MT hijacking will be essential to develop future therapies for its blockade. However, studying this process in real‐world human cancer samples under physiological conditions remains a significant challenge as it is not feasible to label the mitochondria in the tumour infiltrating T cells.

## 2 FINGERPRINT OF MT THEFT IMPLIES TUMOUR AGGRESSIVE PHENOTYPES

**FIGURE 1:**
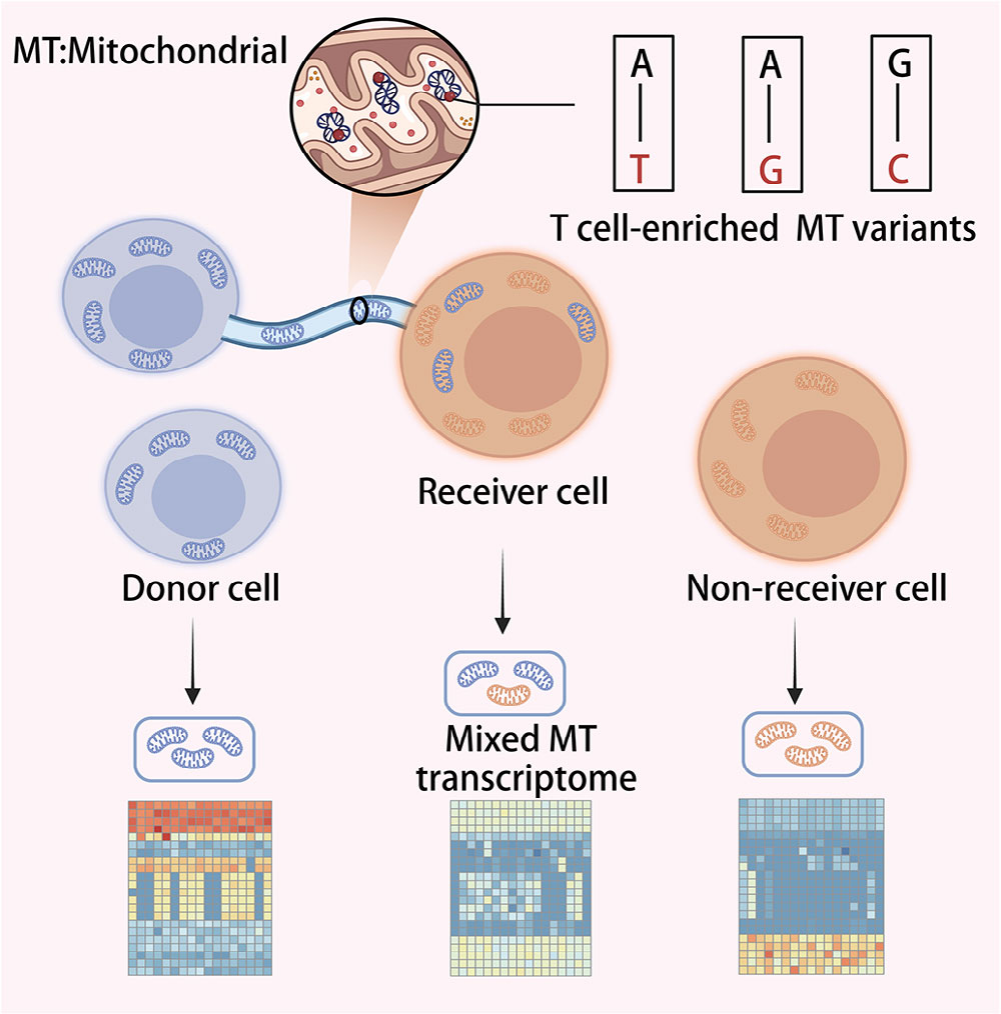
The molecular signatures of mitochondrial (MT) transfer in the receiver cell.

To address this challenge, we developed a novel statistical deconvolution method MERCI.^5^ This algorithm leverages single‐cell genomic data, particularly scRNA‐seq data, to trace the movement of mitochondria between cells. This is achieved by analyzing the MT genome and transcriptome signatures in potential recipient cells (Figure 1). MERCI enables the identification of a subset of cancer cells, termed “MT receivers”, and quantifies the relative extent of MT transfer in each input cell. Our application of MERCI to skin and oesophagal cancer samples revealed that cells identified as MT receivers exhibited higher levels of genes linked to cytoskeleton remodelling and energy production in both cancer types. This discovery reveals a previously unreported cancer phenotype associated with MT transfer. A key future direction is to understand the significance of this mechanism in patients, which requires a comprehensive study across a broad range of cancers. Using MERCI, we defined a group of 17 specific genes implicated in NT formation and MT transfer and created an integrated score—called tumour MT transfer (TMT) score—as a measure of MT transfer in tumours. Analyzing over 10,000 cancer samples from The Cancer Genome Atlas database with this score, we found a strong correlation in various cancer types between increased MT transfer activity and the rapid proliferation of tumours, as evidenced by high cell cycle scores. Additionally, this increased MT transfer was observed to be consistently linked with poorer patient survival across multiple cancer types.

## 3 CLINICAL IMPLICATIONS OF TUMOR‐T CELL MT TRANSFER

Theoretically, cancer cell's ability to hijack energy from immune cells might help explain why some cancer immunotherapies have had limited efficacy. For example, chimeric antigen receptor (CAR)‐T cell therapy, which shows great success in treating certain blood cancers, has faced challenges when applied to solid tumours.^6^ It is possible that CAR‐T cells or endogenous T cells are losing mitochondria due to the tumour's hostile environment, limiting their activity and shortening their lifespan. Genetic signatures reported in our study might guide researchers to design new experiments to see if the presence of MT hijacking correlates with failure from immunotherapies in large datasets. The next key step is identifying the main route that tumour cells use to initiate MT transfer and developing strategies to block this MT theft, to advance MT transfer as a novel therapeutic target. Current understanding, although needs further validation, suggests that intercellular MT exchange primarily occurs through tunnelling NTs (TNTs).^7^, ^8^ Our research identified hundreds of genes that were more active in MT receivers compared to the non‐receivers, and many of these genes are important in NT formation and elongation.^5^ These findings might create opportunities for researchers to either develop drugs that inhibit NT formation or enhance T cell resistance against MT hijacking. Progress in either direction could help scientists take away one of cancer's significant advantages over immune systems and bolster the effectiveness of immunotherapies.

Various strategies could be explored to inhibit MT transfer, with the aim of enhancing the efficacy of cancer treatments.^9^ Reagents like Taxanes and Vinca alkaloids, known for their ability to interfere with microtubule polymerization, have demonstrated utilities in partially blocking MT transfer.^10^ Additionally, the tumour necrosis factor alpha‐induced protein 2 (TNFAIP2), commonly known as M‐sec, plays a crucial role in the formation of TNTs. Targeting M‐sec to inhibit its expression could serve as an indirect strategy to block TNT formation, thereby reducing MT transfer between cells.^11^ While these broad‐spectrum inhibitors have shown limited ability in controlling tumour growth, a search for more specific TNT regulators that can more effectively prevent TNT formation and MT transfer remains necessary.

Another important question under investigation is the timing of MT transfer during the evolution of a tumour. Understanding whether malignant cells started to capture mitochondria from neighbouring cells at the onset of tumorigenesis could lead to novel approaches for early cancer detection, perhaps by assessing elevated exogenous MT levels in suspicious tissues. Since many labs now have the capability to generate single‐cell sequencing data, our method, MERCI, provides a framework for future research to explore these fascinating questions.

## 4 LIMITATIONS AND FUTURE PROSPECT

The current version of MERCI was designed to detect gain‐of‐MT signals in cancer cells. However, its impact on the T cell state after MT transfer has yet to be explored. Prior research indicated that partial loss of mitochondria or repressed biogenesis drives the dysfunction of tumor‐infiltrated T cells (TILs).^1^, ^12^ This new mechanism of MT drainage by cancer cells provided another possible explanation for the metabolic dysfunction and exhaustion observed in TILs. To deeply understand the MT dynamics of the intratumoral T cells, the development of an advanced computational approach is needed to detect the loss‐of‐MT signal in donor cells, thus serving as a complement to MERCI. Furthermore, while MERCI can be directly applied to single‐cell data from human cancer samples, allowing for some level of in vivo investigation, the use of fluorescent dyes and sequencing techniques might require invasive procedures to load cells. These procedures could impact cell survival and might not accurately represent the true physiological conditions within tumour tissues. Hence, new MT labelling or in situ tracing techniques will be ideal to measure MT transfer more realistically in living organisms.

## AUTHOR CONTRIBUTIONS

B.L. supervised the whole writing process and revised the manuscript. H.Z. wrote the manuscript with critical reading and feedback from the B.L.

## CONFLICT OF INTEREST STATEMENT

The authors declare no conflict of interest.

## ETHICS STATEMENT

All authors have been personally and actively involved in substantial work leading to the paper, and will take public responsibility for its content.
